# Limiting medical certainties? Funding challenges for German and comparable public healthcare systems due to AI prediction and how to address them

**DOI:** 10.3389/frai.2022.913093

**Published:** 2022-08-01

**Authors:** Ulrich von Ulmenstein, Max Tretter, David B. Ehrlich, Christina Lauppert von Peharnik

**Affiliations:** ^1^Chair of Public Law, Justus Liebig University of Giessen, Giessen, Germany; ^2^Department of Systematic Theology, Friedrich Alexander University of Erlangen Nuremberg, Erlangen, Bavaria, Germany; ^3^Department of Economics and Management, Karlsruhe Institute of Technology (KIT), Karlsruhe, Baden-Württemberg, Germany

**Keywords:** artificial intelligence, healthcare system (HCS), health insurance, adverse selection, medical certainties

## Abstract

Current technological and medical advances lend substantial momentum to efforts to attain new medical certainties. Artificial Intelligence can enable unprecedented precision and capabilities in forecasting the health conditions of individuals. But, as we lay out, this novel access to medical information threatens to exacerbate adverse selection in the health insurance market. We conduct an interdisciplinary conceptual analysis to study how this risk might be averted, considering legal, ethical, and economic angles. We ask whether it is viable and effective to ban or limit AI and its medical use as well as to limit medical certainties and find that neither of these limitation-based approaches provides an entirely sufficient resolution. Hence, we argue that this challenge must not be neglected in future discussions regarding medical applications of AI forecasting, that it should be addressed on a structural level and we encourage further research on the topic.

## Introduction

Artificial intelligence (AI) is finding its way into more and more areas of life, driving technological change and social development. In medicine, in particular, AI is enjoying great success. For example, the medical use of AI enables more precise diagnoses and allows physicians to determine the best treatment option for their patients (Topol, 2019b; Troisi, 2021)—thus reducing medical uncertainties. In addition, the use of AI leverages evidence-based predictions of individuals' health trajectories and precise determination of their disease risks, i.e., which disease they will most probably contract at what time and how badly (Chaari, 2019; Topol, 2019a)—thus producing new “medical certainties” (Mathews, 1995).

By reducing medical uncertainties, the use of AI can improve medical practice (Pouly et al., 2020). By determining disease risks and producing medical certainties, the use of AI can enable healthcare systems—which are facing ongoing cost explosion and demographic change (Klöckner, 2021; Prasuhn and Wilke, 2021)—to plan more far-sightedly (Wang et al., 2019). If healthcare systems know in advance how many people will contract diseases in the middle to long term future and how many people will need medical treatment, they can hold available the necessary treatment capacities or financial resources, or help prevent those diseases. Thus, by determining disease risks and producing medical certainties, the use of medical AI contributes to improving and sustaining healthcare systems.

On the other hand, however, the medical use of AI may exacerbate existing funding problems in dual public-private healthcare systems (Corea, 2019).^1^ When people know their disease risks with relative accuracy, though never perfectly, and have access to high degree of medical certainties, there is a risk that people with favorable health trajectories will switch from statutory health insurance—where they have to pay a premium based on individual income—to private health insurance—where their premium is based on individual risk—while people with overall unfavorable disease risks will stay in or switch to statutory health insurance. Such dynamics, known as “adverse selection” (Akerlof, 1970), challenge the funding of statutory health insurance (van Kleef et al., 2020).

This scenario raises the question of whether and how to counteract what others have called “the threat of adverse selection” (Jong, 2021). Taking an interdisciplinary approach, conducting conceptual analyses, thought experiments, and legal as well as moral assessments, we aim to answer this question in our paper. In particular, we will focus on the question if it is possible to counteract adverse selection by limiting the use of AI in medicine and establishing limits for medical certainties. However, we find that it is not possible to counteract adverse selection by using only limitations—and that instead it is necessary to rethink healthcare systems and their distinction between public and private healthcare systems.

To support our argument, we first conduct a conceptual analysis and show how the use of AI can, to a certain extent, reduce medical uncertainties and create new medical certainties by determining disease risks. Performing thought experiments, we then show how these new certainties can exacerbate adverse selection and challenge the funding of the statutory health insurance. We focus on the example of the German healthcare system, where certain individuals can choose whether to be part of statutory or private health insurance. After showing that existing safeguarding mechanisms are not sufficient to counteract adverse selection, we ask whether it is possible to counteract adverse selection by introducing limitations to the medical use of AI. In a legal and moral assessment, we will present four possible limitations and show why they are not feasible or not able to achieve this goal. In an interdisciplinary discussion we show that adverse selection can only be counteracted by fundamentally rethinking the structure of dual healthcare systems and reforming the distinction between statutory and private health insurance. We will then identify some limitations before summarizing our findings in a conclusion.

## How does the use of medical AI produce new medical certainties?

Medicine is currently undergoing a process of pervasive digitization. Telediagnoses, electronic health records, remote surgery, as well as the introduction of digital twins are but a few among many examples of the novel opportunities. These new technologies promise to make medicine more accessible for many people (Flores et al., 2013) while also enabling a more personalized approach toward medicine (Chaari, 2019). The latest step is the introduction of AI into medicine (Topol, 2019a).

By AI, we mean self-learning algorithms trained with large amounts of data (so-called Big Data), to recognize patterns and draw conclusions from them. In recursive processes, the algorithms are given feedback on whether their conclusions are correct. They then use this feedback to learn, i.e., to rewrite their codes and improve their conclusion-making capabilities. Due to their computational power and learning abilities, AI can outperform humans in recognizing patterns in large amounts of data after only a short period of learning (Boden, 2018). That's why AI is used in many fields where large amounts of data need to be analyzed and evaluated.

Exemplary for the introduction of AI in medicine is its use in dermatology, tumor board conferences or for public health surveillance (Schwalbe and Wahl, 2020). Dermatologists usually build on many years of training and practical experience in classifying skin irregularities either as harmless or potentially dangerous. AI however, due to its automated image processing, is able to analyze images within seconds and compare vast databases of similar cases. This allows AI to determine skin irregularities faster than any dermatologist, and often with higher precision and less errors (Du-Harpur et al., 2020; Pouly et al., 2020). In another application, AI is used in tumor board conferences, where health professionals—often from diverse disciplines and with their respective expertise—get together to discuss available options for a patient's cancer treatment (Somashekhar et al., 2018). While even the most experienced physician has limited memory and reasoning capabilities, AI analyzes a patient's data, identifies relevant patterns, compares the individual case to many other cases, their treatments and outcomes, and thus provides treatment recommendations that help health professionals in these conferences make informed decisions (Bleher and Braun, 2022). A third possible application of medical AI is public health surveillance (Chiolero and Buckeridge, 2020). Here, AI technologies are applied to monitor the outbreak and/or spread of infectious diseases using various data sets, including official disease data, social media data, as well as individual movement and contact data. This AI-produced information not only reduces public health uncertainties about the spread and infectiousness of diseases, but can also help to find efficient countermeasures, to model and assess public health responses—and thus, at best, prevent further pandemics (Zeng et al., 2021).

In addition to reducing medical uncertainties in acute medical care or public health, AI produces new medical certainties by predicting with unprecedented precision, how people's health condition will develop. A case at hand is the use of digital twins—digital simulations of real persons based on their health-relevant biomedical (e.g., heart rate, blood oxygen saturation, blood pressure) and lifestyle data (e.g., exercise, physical activity, sleep cycles, consumption patterns, diet). This data is constantly updated by sensors in real time and modeled by an AI to create dynamic *in silico* simulations of persons—their digital twins. Even though digital twins are still in their infancy and are currently used primarily for research purposes, a wide range of studies are testing how they can be used medically (Ahmadi-Assalemi et al., 2020). For example, there are current studies in which digital twins are used to test in a virtual simulation how well a patient reacts to different drugs and which has the best efficacy (Björnsson et al., 2020). In other studies, digital twins are being used to perform virtual surgeries on a specific person and simulate whether and how he tolerates them and what benefits they would have for him (Ahmed and Devoto, 2021).

Further, by taking into account all data and extrapolating past and current health trends, digital twins promise fairly precise personalized predictions, e.g., determining the statistical risk of falling ill with particular diseases (Hafez, 2020) within a certain period of time with a high degree of accuracy (Huang et al., 2022). The latter can eventually span the entire lifespan and thus could allow for a complete profile of persons' health and their individual disease risks.^2^

Of course, neither a digital twin nor any other AI is currently capable of predicting a person's health trajectory with absolute accuracy or determining their disease risks beyond a shadow of a doubt. In fact, given various technical limitations of AI, e.g., incomplete or biased data sets, (unintentionally) discriminatory or opaque algorithms, it seems likely that AI predictions will *never* be able to provide 100% certainty about a person's health. But even though AI may never provide absolute certainty, the (limited) certainties it *can* produce are expected to have an enormous impact on the behavior of physicians or patients. For example, even diagnoses that are only 80% certain or treatment recommendations that are only 75% optimal will further help physicians make decisions and act. Similarly, it is likely that even a very good or a very bad health prediction, even if it is only so-and-so certain, will influence people's decisions. Especially if we assume that research about AI in medicine will continue to make progress in the coming years and decades—if recent and current trends in AI-research prove robust (Pouly et al., 2020)—and that AI-technologies will become ever better and more precise at determining their disease risks of people and predicting their health trajectory in the future, we have to assume that people will be increasingly influenced by these AI-produced certainties.

These new medical certainties, as well as the fact that individuals align their decisions and actions with them, although neither is ever completely certain, hold great opportunities. Equipping physicians and patients with highly accurate predictions about their health status and determinations of their disease risks for example, will enable earlier detection and treatment—even before a person falls ill. Ideally, diseases can be prevented completely because of very early measures taken and personal suffering can be spared (Vaishya et al., 2020). It also presents itself as an exciting opportunity for healthcare systems. By predicting how many people will contract diseases in the future and will need medical treatment, these new medical certainties can enable healthcare systems to plan more far-sightedly and safely (Panch et al., 2018; Schwalbe and Wahl, 2020). They can free up or create the necessary treatment capacities and financial resources—and withdraw them where they do not need them. Thus, the medical use of AI can mitigate prevalent cost pressures and sustain healthcare systems (Knorre et al., 2020). In all these sectors, AI can and will increasingly take over functions ranging from pure data analysis and decision support to the complete assumption of decision-making. This does not necessarily mean that human actors like physicians will disappear from the medical field nor that this is the aim of this process (Topol, 2019a; Araujo et al., 2020).

Nevertheless, the influence is so immense that fundamental restructuring is to be expected. Correspondingly, the new medical certainties generated by AI also raise challenging questions. One first question is how certainty about one's own future health development affects people, their self- and world perception as well as their psyche and life planning—especially if AI predicts unfavorable health developments (de Boer, 2020; Tretter, 2021). A second question is, how these effects on people have a further impact on (health) policy making (Margetts and Dorobantu, 2019; Coeckelbergh, 2022). Another question of primary concern to us is how increasing medical certainty will affect healthcare systems and their funding. In particular, the medical use of AI may exacerbate adverse selection, where individuals with low risk of disease migrate to private health insurance—which might lead to funding problems for statutory health insurances.

## How do new medical certainties challenge healthcare systems?

We focus on the second question and outline the challenges that new medical certainties pose for healthcare systems, we will first describe how healthcare systems are funded on a solidarity basis. We focus on the example of Germany with its dual healthcare system of statutory health insurance on the one, private health insurance on the other hand and the option to switch between the two. While both types of insurance systems in principle offer similar insurance against health related cost risk to their policyholders, they mainly differ in how they calculate their premiums. After describing this difference and considering which form of insurance is of interest to whom, we present how new medical certainties can lead to adverse selection—and how this challenges the funding of statutory health insurances.

### Healthcare system in Germany—statutory and private health insurance

Almost all healthcare systems in the Western world are funded on a solidary basis (Rice, 2021; Schölkopf and Grimmeisen, 2021), i.e., their members pay a regular premium into a common insurance pool. If a person falls ill, the financial cost of recovery, regeneration and other cost is paid from this common insurance pool. By covering this cost through their regular premiums, the members of the healthcare system in the end support each other in solidarity.

In the German healthcare system, the way of calculating the individual premiums diverges between statutory and private health insurance. The premiums in private health insurance are typically calculated on the basis of risk of disease (Müller-Peters and Wagner, 2017). If a person has a high risk, she has to pay higher insurance premiums, with a low risk lower insurance premiums. The individual disease risks of persons are determined by private health insurance companies on the basis of certain statistical and behavioral data (Igl and Welti, 2018)—for example, pre-existing conditions, age, and if they smoke (Albrecht, 2018; Hoffmann, 2021).

The premiums in statutory health insurance instead follow two subsidization mechanisms. First, the insurance premium is not calculated according to the individual risk of disease, i.e., people with high risk do not pay higher premiums than people with low risk of disease. The reason for this “subsidizing risk solidarity” (Lehtonen and Liukko, 2011) is to avoid placing the extra burden of high insurance premiums on individuals with a high risk of disease. Instead, insurance premiums are based on individual income, i.e., people with high incomes pay higher premiums than people with low incomes. The reason for this “subsidizing income solidarity” (Lehtonen and Liukko, 2011) is to prevent, that people with a low income have to pay a large share of their income for insurance contributions, while people with a high income have to pay only a small part of their income for insurance contributions. In principle, policyholders currently have to pay 14.6% of their contributory income to their GKV (§ 241 SGB V). If a statutory health insurance is not allocated enough funds, for example because it insures too high risks, it must levy its own additional contribution to fill this gap (§ 242 SGB V). Beyond that, there is no regulatory framework linking the level of disease risk and the income-based premium.

Most Western countries with the exception of the United States have public healthcare systems and citizens are required to participate in them (Rice, 2021; Schölkopf and Grimmeisen, 2021). In almost all of these countries it is possible to contract *additional* private health insurance for services not covered by public healthcare. In some countries, however, citizens also have the option of opting out of public healthcare systems and instead acquiring private health insurance exclusively. This, for example, is possible in Germany, where currently 88.2% of the citizens are part of the statutory health insurance, its public healthcare system (GKV-Spitzenverband, 2021) and only 5.2% are insured exclusively with private health insurance.

### Interests of policy holders

Healthcare systems produce a benefit for their members. The person is financially protected in the event of disease and does not have to pay by herself for occurring cost. Likewise, being part of a healthcare system generates cost for individuals in the form of their premiums. People weigh financial cost and benefits, among other factors (Richter et al., 2019), in order to identify the optimal choice when they are seeking insurance since it is in their rational self-interest to pick the contract which provides them with the largest overall benefit (Corea, 2019). Since, as noted above, the benefits in statutory and private health insurance are roughly the same—both offer basic health coverage, private health insurance offers some additional benefits (§ 11 SGB V, § 192 VVG)—it is mainly the cost that is decisive for the choice between the two types of insurance (Lünich and Starke, 2021).

For this choice policyholders will look at their individual disease risks and compare how much they have to pay in different insurance systems. Individuals with a high risk of disease—potentially, for example, those with pre-existing conditions or diseases—might take into account that they pay more in private health insurance, where premiums are calculated based on individual risk. In turn, individuals with low risk of disease may see that they have to pay less in private than in statutory health insurance.

As long as the individual risk cannot be determined precisely, i.e., as long as there is uncertainty about the individual risk, the choice between statutory and private health insurance is kind of a “gamble”—both for the individuals and for the institutions. Neither individuals nor insurers know the exact person's risk, i.e., how much cost they will approximately generate and how high their premiums must be to cover this cost (Jong, 2021). This phenomenon is not limited to medicine, it is also known in other fields, like finance.

**Figure 1:**
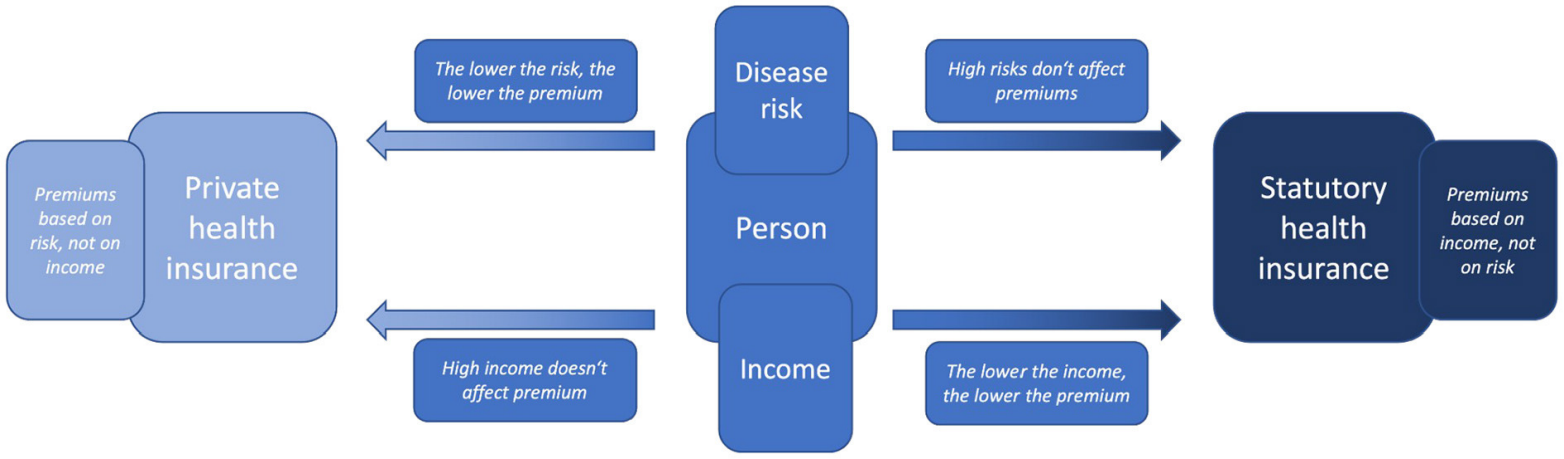
Illustration of the choice an individual may face between either statutory or private health insurance and the according price determinants income or health risk (created by the authors).

Now, if AI makes it possible to predict a person's health trajectory and accurately determine her risks and if many people have access to AI and its predictions, this medical uncertainty gradually fades away. This can have effects on the person's decision between statutory or private health insurance—already without AI being intended to replace decisions. This is because people generally have confidence in the fairness and usefulness of AI-assisted decisions, even if they are of high impact. In fact people often evaluate these decisions taken by AI par or even higher in comparison to human experts (Araujo et al., 2020). Now if AI predicts that a person's health will develop unfavorably and that she has a high risk of disease, she may obtain the certainty that due to her risk and income it would be financially more favorable for her to get statutory health insurance. Conversely, if AI predicts that a person's health will develop favorably and that she has very low risk, it would be advantageous for her to be privately insured and to pay an individualized premium (Figure 1).

This can be illustrated using a simplified example—simplified as it considers only the risk of one disease and not the cumulative risk of all diseases as would be relevant for healthcare systems. For example, suppose an AI were to calculate a 25% risk for a middle-class male in his late 20s living in Germany, to develop cancer during his lifetime. His risk would then be significantly lower than the 47.5% average lifetime cancer-risk for an average German male (SwissLife, 2020). His significantly lower disease risks would mean that his risk-based premium in private health insurance would also be very low. Now, if he did not earn so little money that his income-based premium of statutory health insurance would be even lower, having low disease risks would be a strong incentive to choose private health insurance. However, if the AI did not diagnose him with a 25% risk, but with an 80% risk, this would have the opposite effect on his choice. As his risk of illness increases, his risk-based premium in private health insurance would also increase significantly. Now, if he did not earn so much money that his income-based premium of statutory health insurance would be even higher, his high risk of disease would be a strong incentive to choose a statutory health insurance. However, the latter is hardly possible in Germany as the so-called “Beitragsbemessungsgrenze” sets a limit for the amount of income considered for calculation [§ 223 *Section* 3 SGB V (Federal Ministry of Health, 2020)]. Any income above 58,050 Euro in 2022 (Bundesregierung, 2022) must not be considered in income-based premium calculations. Using the example of cancer, it is illustrated how differences in income and (cumulative) disease risks can affect a person's premium as well as her choice between private health insurance (and their risk-based premiums) and statutory health insurance (and their income-based premium).

### Adverse selection and funding issues for public healthcare systems

When individuals obtain new medical certainties regarding their individual disease risk in relation to their expenses for insurance premiums (which are already certain information to them), this enables them to perform a cost-benefit assessment that is more precise than before. The opportunity can be beneficial for “low risk” individuals. If their disease risks are below average, they might have an incentive to switch to private health insurance where they might be able to get insurance for lower premium payments after revealing their low risk profile. For “high risk” individuals already in the private health insurance, on the other hand, the new medical certainty might be a concern. They can expect a significant increase in their individualized premium rates if the private insurer obtains access to the medical information as well.

The novel possibilities of precisely estimating individual risk can also pose a major challenge for the statutory healthcare system as a whole. That is the case for countries—like Germany—where people are able to opt out of the public healthcare system and instead obtain private health insurance. A significant portion of (low risk) individuals would have an incentive to take advantage of this option, and to get private health insurance in order to save money. Likewise, “high risk” individuals in private health insurance have an interest to switch to statutory health insurance. This effect triggers a splitting development: Individuals with higher disease risks remain in, or switch to, the statutory health insurance while individuals with lower disease risks move toward the private health insurance or remain privately insured if they are already. This phenomenon is an incidence of “adverse selection” (Bitter and Uphues, 2017). In the decades following George Akerlof's seminal “lemons” paper (Akerlof, 1970)—representative for the work that led to him receiving the 2001 Nobel Prize in Economic Sciences—economists have thoroughly studied the issue (Browne, 1992; Simon, 2005) also with regard to health insurance. Since asymmetric information is *the* underlying factor for adverse selection, and since asymmetric information is usually highly prevalent in insurance markets, the issue bears special significance for these markets. Empirical studies have pointed out how adverse selection may generate societal costs and welfare loss (Cutler and Reber, 1998).

In the particular case of statutory health insurance, adverse selection is a concern as it might gradually shift the equilibrium between “low risk” and “high risk” individuals toward a higher average risk. When the average risk (per insured person) increases, so does the average expected cost (per insured person). Since the insurance system must cover its expenses and balance its books in the long run, this leads to rising premiums. When premiums rise, this can set in motion a self-reinforcing feedback loop (or *vicious cycle*), as the higher premiums mean that, now, an even larger share of people has an incentive to opt out and the cycle begins anew. In a similar context, Cutler and Zeckhauser (1998) used the term “adverse selection death spiral” to describe such a potential development.

With respect to funding issues of statutory health insurance (Bitter and Uphues, 2017), it is further important to keep in mind that “low risk” individuals pay—on average—more into the statutory health insurance than they get back in covered medical cost. Thus, the health insurance receives a net revenue gain from “low risk” persons which enables the insurer to subsidize high-risk persons who generate more cost than revenue (as their risk is higher than the one they pay for). To counteract the loss of revenue due to adverse selection, statutory health insurance must thus raise premiums significantly which jeopardizes the purpose of statutory health insurance, i.e., to safeguard general access to medical treatments especially for poor or vulnerable individuals (Prasuhn and Wilke, 2021). If the vicious cycle is allowed to continue unopposed, this would have the potential to ultimately derail the whole funding of the health system, since premiums might increase to unprecedented heights and the group of people able to pay them would continuously dwindle.

**Figure 2:**
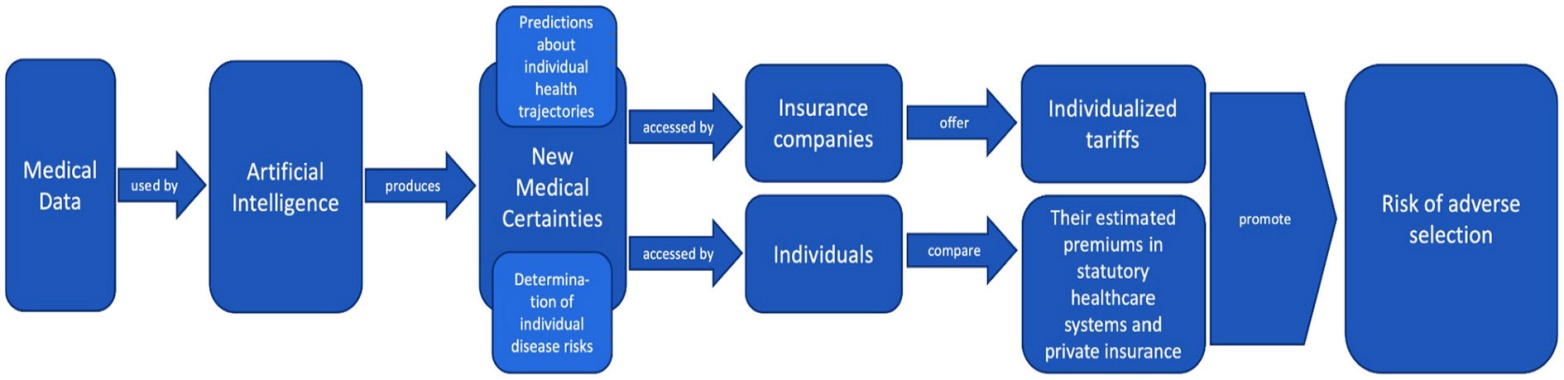
Illustration of how AI produces new medical certainties from medical data, how these certainties are used by insurance companies and individual policyholders, and how this ultimately exacerbates adverse selection (created by the authors).

This process of how AI produces new medical certainties from medical data and how this ultimately exacerbates adverse selection can be illustrated in Figure 2.

## Why are current tools not sufficient to counter adverse selection?

As the previous sections have shown, AI produces new medical certainties by determining individual disease risks. These new medical certainties enable people to weigh the cost and benefits of both types of insurance systems more precisely and to choose between statutory and private health insurance. This possibility bears the risk that persons with low risks opt out of statutory health insurance and choose private health insurance only. Adverse selection poses a threat to statutory health insurance, as it jeopardizes their funding in the long term.

Within the healthcare system in Germany, there are some mechanisms that might be able to counteract adverse selection and cushion the challenge it poses to the funding of statutory health insurance. Those mechanisms were not designed to specifically tackle threats due to the use of AI in medicine, but to safeguard and sustain reliable funding.

First, not every person is free to sign a contract with private health insurance. Citizens are generally obliged to have health insurance (§ 193 VVG) while most of them are obliged to take out a statutory health insurance (§ 5 SGB V). Only certain circumstances exempt people from this obligation and allow them to acquire private insurance exclusively (§ 9 SGB V). The exempting criteria include an income threshold-−64,350 Euro in 2022. It also applies to civil servants, clergy persons and full-time self-employed individuals. The Federal Ministry of Health is counting on a 10.4% share of voluntarily insured members in the statutory health insurance in 2021 (GKV-Spitzenverband, 2021). According to the Federal Ministry of Social Security, however, the share of contributions to statutory health insurance paid by voluntarily insured members will be 21.3% in 2021 (Bundesamt für Soziale Sicherung, 2021). That is, while only these people have the option to switch insurance, they, in particular, contribute disproportionately much to overall funding of statutory health insurance.

Furthermore, private health insurers are subject to various legal principles that, at least partially, impede insurance premiums from being exclusively based on individual disease risks and to offer individualized so-called *pay-as-you-live-tariffs* (Bitter and Uphues, 2017; Brömmelmeyer, 2017; Albrecht, 2018; Hoffmann, 2021). Without an offer of an individualized insurance premium, there is less incentive to leave the statutory health insurance. As a consequence, there is less leeway for adverse selection.

By limiting how many people can switch and how much insurance companies can individualize their tariffs and premiums, legal regulations can counteract adverse selection to a certain extent. However, they can never prevent adverse selection, as they cannot stop private insurers from determining individual risks or offering risk-based tariffs. Rather, the latter is their legally mandated task [§10 KVAV (Albrecht, 2018)].

This shows existing mechanisms to be sufficient to attenuate some short-term adverse selection. These mechanisms are, however, not able to cushion or counteract widespread and persistent adverse selection, with large proportions of people with low disease risks opting for private health insurance and large proportions of high-risk people opting for statutory health insurance. Unfortunately, it is exactly the latter that poses enormous long-term threats to the funding of statutory health insurance and calls for other mechanisms to counteract adverse selection.

## Using bans or limits to counteract adverse selection?

One way to counteract adverse selection could be to prohibit the medical use of AI. Several open letters (Conn, 2017), governance papers (Datenethikkommission, 2019), and regulation drafts (European Parlament European Council, 2021), discuss banning research on and use of AI in other fields where it is considered to be too dangerous. So far, however, such demands have primarily referred to military AI or “certain AI systems intended to distort human behavior, whereby physical or psychological harms are likely to occur” (European Parlament European Council, 2021). Regulation targeting medical use of AI has been proposed in China (De Wei, 2021): The draft law “Announcement on Public Comments on the Detailed Rules for Internet Diagnosis and Treatment Supervision” in Article 13 aims to significantly restrict the use of AI for internet diagnoses of patients (National Health Commission Medical Administration Hospital Administration, 2021).

Adapting a similar approach could prevent AI from producing medical certainties and determining disease risks that in turn enable adverse selection. Such prohibition would, however, also prevent medical improvements, inhibit technological progress and would thus constitute a handicap in international competition between states (Oh et al., 2021). Furthermore, medical certainties can also be created without the use of AI, e.g., by making use of genetic testing (Paul et al., 2014). Ultimately, banning such useful technologies seems problematic from the perspective of liberal and democratic states, making it a rare exception that can only be considered for extreme cases, such as the editing of the human germline genome (Boggio et al., 2019).

Rather than completely banning medical use of AI, one might think about *limiting* the medical use of AI. A German approach on limiting AI in healthcare would have to be integrated into both the German and the European AI strategies. Both strategies provide for strong support of AI research, also and especially in the healthcare sector (European Commission, 2021; Bundesministerium für Gesundheit, 2022). The EU has recently presented a draft on the general regulation of AI, which centrally provides for a risk assessment of AI uses. This assessment leads to a ban or to certain framework conditions for the use of AI (European Parlament European Council, 2021). On the one hand, this conditions refer to the input level and provide for quality standards for the AI training data. On the other hand, they refer on the output level and provide that the results of AI systems should be verifiable (Ebert and Spiecker gen. Döhmann, 2021). Overall, this draft has been positively received by Germany (Deutscher Bundestag, 2022) and although the regulatory project has not yet been finalized it seems like both, Germany and the EU, are walking the same way on implementing AI in healthcare system. Nevertheless, this approach is recognizably at odds with our critical analysis of medical certainties, and is more likely to promote them, as it primarily refers to conditions which promote more precise predictions of AI. Accordingly, the approach of limiting certainty-producing AI can complement the regulatory approaches of the EU and Germany. The desired goal would be to limit AI in a way that allows it to continue to produce medical certainties that are useful for medical practice, but prevents it from producing a high degree of medical certainties that could exacerbate adverse selection. Since this is a rather non-specific idea so far, the question from the perspective of a regulating entity is: which regulatable aspect of AI could be limited in order to counteract adverse selection without rendering AI completely useless for medical purposes? In this chapter, we will therefore discuss the four most promising aspects that appear to be causal for the occurrence of adverse selection and thus have the potential for limiting regulation.

### Limiting the computational power of AI

A starting point would address the technical framework. A key technical aspect behind the great potential of AI is its underlying overall computational power: the more processes an AI system can perform in a given period of time, the more data it can analyze and the more accurate predictions it can theoretically make. Higher computational power has the potential to create more medical certainty, which, as described, favors adverse selection. By limiting the computational power of medical AI, one could prevent it from generating a high degree of medical certainty and thus counteract adverse selection (Hwang, 2018).

However, a closer look reveals that the presumed direct correlation between computing power and medical certainty exists only loosely. Dermatological analysis *via* smartphone app shows that for special tasks comparatively little computational power can produce high levels of medical certainty (Topol, 2019b). Conversely, AI equipped with immense computing power but not operated by expert persons or is equipped with insufficient data (e.g., too little or too inaccurate) might produce little to no medical certainty.

Tackling adverse selection by limiting the computational power of medical AI would be pointless at best and harmful at worst. It would be pointless because trained personnel with good data could produce high levels of medical certainty, even with little computational power. It would be harmful if the limitation of computational power—despite good data and the use by trained personnel—would lead to inaccurate prognoses, stand in the way of the success of medical treatments and thus endanger human lives.

### Limiting the output of medical AI

Instead of addressing technical frameworks and limiting the computational power of AI, another approach would focus directly on the output of AI (Swedloff, 2014). This would avoid the problem of regulation overlooking aspects that are relevant to producing medical certainty or, conversely, overemphasizing irrelevant aspects.

If the AI were to produce levels of medical certainty that did not carry the risk of exacerbating adverse selection—e.g., because the person's high individual disease risks would not correspond with low premiums in private health insurance (Swedloff, 2014), its medical use would be permissible. Conversely, if the AI were to produce a level of medical certainty that might exacerbate adverse selection—e.g., because the person's low individual disease risks would correspond with low premiums in private health insurance, it may not be used for such risk. Accordingly, such an order would be rendered absurd by the certainty already produced. At first glance, this seems to be an appropriate starting point for regulation. However, the content of the regulation is not technically feasible.

A limitation of certainty in the constellations just described, which show a risk of adverse selection, could be that AI may not be used for such risk predictions. However, this paradoxically requires medical certainty to be produced first in order to subsequently allow banning this production of certainty.

### Limiting access to medical certainties

A third option would not try to set a limit on AI or its medical use but would rather focus on the medical certainties produced by it. If these certainties could exacerbate adverse selection—e.g., if the individual disease risks would correspond with low premiums in health insurance, the patients' access to them could be limited. While physicians might still be allowed to access these medical certainties and use them for treatment purposes, the patients themselves might not be granted access to their medical certainties. This would prevent them from updating their assumptions regarding their individual disease risks and leaving statutory health insurance.

However, this approach appears paternalistic and not in line with a liberal society. After all, people have a fundamental right to obtain their own medical information. To restrict this right would constitute a legal novelty and require sound justification. To find such justification might turn out to be difficult since one's own medical information is rather close to the very core of personal rights. After all, if information about her medical risks is withheld from the patient, the patient may become suspicious and draw conclusions about her level of risk.

Finally, we can expect to see private companies offering direct-to-consumer AI based medical prediction—similar to *23andme* and *Ancestry* business models in the context of genetic testing (Thiebes et al., 2020). Given these direct-to-consumer opportunities, it will be difficult to limit individuals' access to medical certainties.

### Tying access to certainties to agenda-driven conversations

A fourth approach to counteract adverse selection would acquaint patients with the advantages of statutory health insurance over private health insurance and also appeal to their individual solidarity. This could take place during the physician-patient conversation, when patients are informed about their medical risks and certainties. The physician could appeal to the conscience of the patients (Moloi and Marwala, 2020), point out the high value of the solidarity-based nature of statutory health insurance or show how paying individual one's premiums helps to save lives and preserve the quality of life of others. This approach could even be legislated in the form of a general duty to inform, requiring physicians to communicate medical risks and certainties to their patients exclusively in a conversation that must include the above directions and appeals.

There are several problems with this approach. First, it places the responsibility on physicians to prevent adverse selection. This represents a massive non-specialist overload on physicians. It risks undermining the trust relationship between doctor and patients as the patients could no longer be certain whether a doctor has only decided exclusively in their best interests or if a doctor actually bears diverging objectives in mind. Furthermore, this approach would be an attempt to solve a structural problem on an individual level—which appears to be unsustainable.

## Discussion

**Figure 3:**
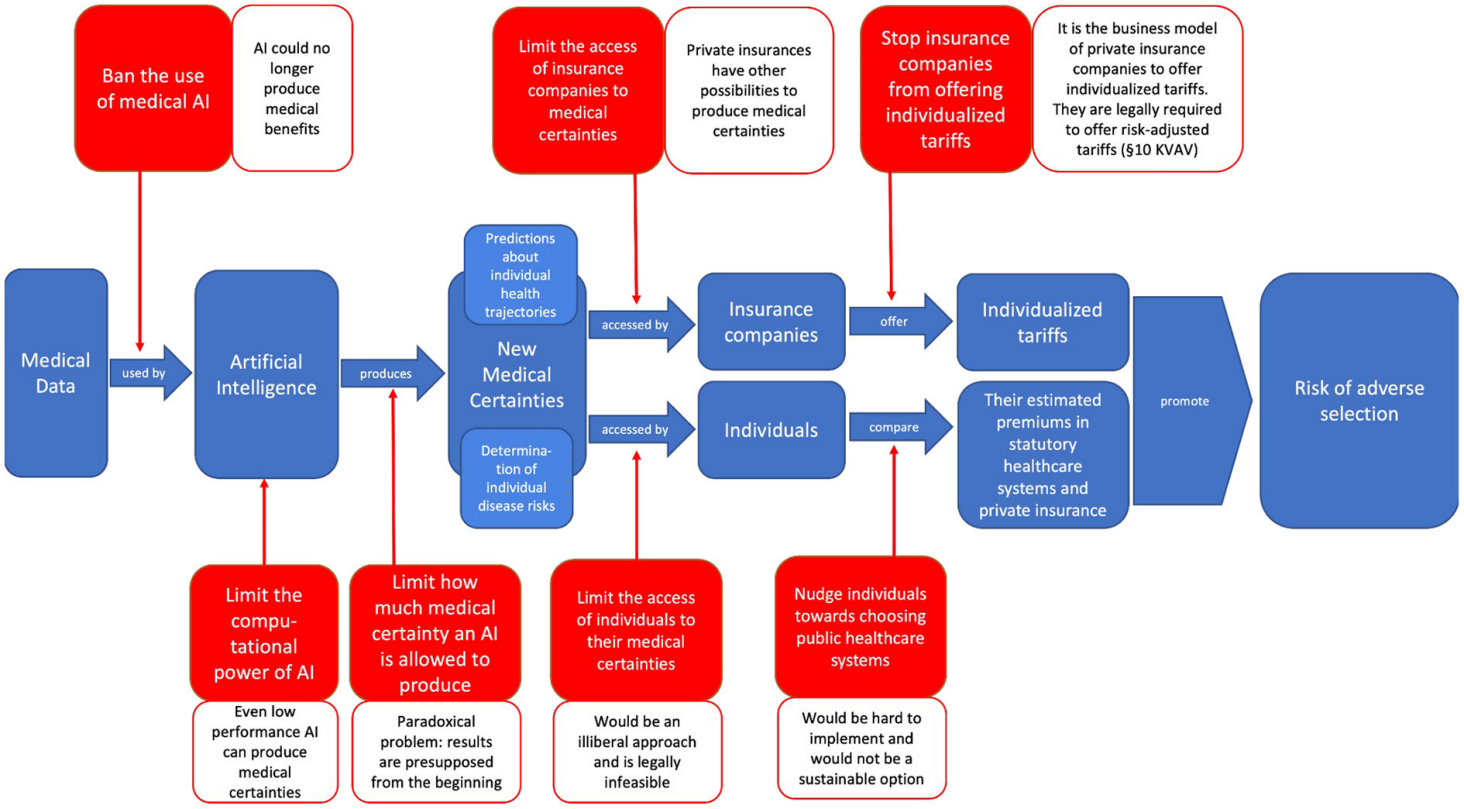
Illustration of the different possibilities of banning and limiting medical AI or the access to or use of medical certainties in order to stop them from exacerbating adverse selection—as well as the different reasons why these options fail to do so (created by the authors).

We have shown that neither banning or limiting AI and its medical use nor limiting the access to or use of medical certainties seems to be a viable approach to counteract adverse selection. The different possibilities of banning and limiting, as well as the reasons why they fail to counteract adverse selection, can be illustrated (Figure 3).

As all discussed limiting options fail to solve adverse selection, policy-makers might want to consider more comprehensive approaches. After showing that adverse selection is a fundamental problem of the dual public-private healthcare system and does not arise only as a result of AI, we will propose in this chapter that it can only be counteracted at a structural level—by rethinking healthcare systems' dual nature. In Germany, there are already some debates on whether and how to restructure the national healthcare system. After introducing these discussions and briefly showing how our results may contribute to them, we will ask for whom our considerations are relevant—only for the German context or also for the healthcare systems of other countries—and survey empirical evidence to validate our theoretical discussion.

### Ongoing debates about structural reforms of the healthcare system

We have assumed medical AI to produce certainties that can *exacerbate* adverse selection in statutory health insurance. This wording indicates that adverse selection is not generated by the use of medical AI in the first place. Instead, adverse selection is a general problem of dual healthcare systems, where individuals have the option to switch between statutory and private health insurance (Cutler and Zeckhauser, 1998). Thus, to effectively address adverse selection in the healthcare system, and not just counteract the factors that promote it, it might be most promising to address the problem at a structural level and think about how to reform the healthcare system (Albrecht, 2018)—and not make the mistake of focusing solely on regulating AI or new certainties.

In Germany there are longstanding political debates regarding the question whether and how to reform its current healthcare system. One prominent proposal is discussed under the label “Bürgerversicherung.” It calls for a unified healthcare system in which all citizens participate and equally share the cost of their common disease risks, which would ensure the basic provision of medical services for all citizens and in which there is no possibility of opting out (Prasuhn and Wilke, 2021). While there would still be the option of obtaining additional private health insurance—thus retaining a degree of personal freedom of choice for citizens (Hussey and Anderson, 2003)—this proposal would dissolve the dual structure in which citizens can alternatively switch between public and private healthcare systems.

Since adverse selection is a fundamental challenge especially of dual healthcare systems (Prasuhn and Wilke, 2021), switching to a single-payer healthcare system would tackle adverse selection at the structural level. And if adverse selection is no longer possible, there is no longer the threat that using AI in medicine may exacerbate it. In a single payer system, it would be possible to apply AI to medicine, use its predictive capabilities to the maximum, and enjoy its benefits—better treatment and prevention options for patients, more predictive and safer planning and resource allocation options for healthcare systems, as well as lower insurance premiums for the individuals (Corea, 2019)—without having to worry about or having to take into consideration side-effects on incentives for adverse selection. These upsides remain robust across a variety of concrete configurations of the single payer system. One might, e.g., conceive of a more comprehensive approach to determining individual premiums which considers both risk and income of a person and might concurrently produce a surplus in efficiency. At the same time, some questions concerning the medical use of AI remain unaffected by switching to a single-payer system: how much certainty produced by AI is desirable in itself, how certainty about their future health affects peoples' self- and world perception, or whether in a single-payer system they can help to avoid treatment cost that seem superfluous through preventive behavior of the insured (Swedloff, 2014; Albrecht, 2018)?

In summary, we show how a single-payer healthcare system could enable the full use of AI in medicine and, conversely, how the use of AI in medicine could help make these healthcare systems more efficient and sustainable. Thus, without taking clear positions for or against such structural reforms, we provide some further arguments that might prove helpful in these discussions.

### Scope of our results

While considering the medical use of AI and adverse selection, we focused mainly on Germany, its dual healthcare system and the legal situation there. Germany proved to be a good example, as the risk of adverse selection is particularly high, due to the reasons mentioned above.

However, our considerations are not limited to Germany. Rather, precisely due to the abstract nature of the concept of adverse selection, our considerations prove transferable to all contexts in which there is the possibility of individuals opting out of public healthcare systems and obtaining private health insurance exclusively—in short, to all countries with a dual private-public healthcare system. Besides Germany, these include Austria, France, Belgium, Luxemburg, and Japan (Rice, 2021; Schölkopf and Grimmeisen, 2021). However, these considerations also prove to be fundamental for the United States, where there are exclusively private health insurers and state welfare institutions—because, as evidence hints (Cameron and Trivedi, 1991), for people who are not part of the latter, their medical certainties might prove relevant for choosing private health insurance or no health insurance at all.

In this development, it is obvious that different AI systems will be established in different fields of medical application. Likewise, not all AI systems and the certainties they produce will be accessible right away. However, the technical framework strongly suggests that overall, and despite these differences in access and application, the level at which AI reduces uncertainties and produces certainties will increase.

### Need for further empirical research

Further research on the effects of AI-based health prediction and new medical certainties on adverse selection in the healthcare system will have to elicit empirical data on the matter in order to realistically evaluate and specify these rather theoretical considerations.

Despite our paper being limited by not presenting empirical evidence, our considerations are in line with behavior that has been studied extensively and for a long time. Even though people have been found to exhibit social preferences (Kahneman et al., 2000; Fehr et al., 2008), and monetary incentives not being the only relevant factor in consideration (Andreoni, 1989; Regner, 2015), financial motives play a central role in decision-making. Also people tend to trust in the decision-making-ability of AI (Araujo et al., 2020). Which is why we assume, that people will also trust in the predictions given by AI.

There are empirical studies on related issues that may hint at initial empirical evidence supporting our considerations on adverse selection. Lünich and Starke (2021), e.g., investigated (in a not yet peer-reviewed study) to what extent personal financial benefits influenced persons' attitudes toward dual healthcare systems and their choice between public and private healthcare systems. They conclude that financial benefits, or the prospect of them, may have considerable influence on individual attitudes and decisions, as individuals often express their intent to switch to private health insurance if they expect to gain a financial benefit from it. Other studies have investigated the influence of fitness- and health-wearables on individual solidarity attitudes. They find people using wearables to be more likely to show less solidarity with other people and conclude that the digitization of health can be a challenge for public healthcare systems (Böning et al., 2019; Maier-Rigaud and Böning, 2020). Other studies have begun to empirically examine people's willingness to disclose private data in exchange for monetary benefits (Beresford et al., 2011; Schudy and Utikal, 2017). The above-mentioned studies and their results give no reason to doubt our theoretical considerations on medical certainty and adverse selection. On the contrary, they suggest that our considerations are valid—even if further studies on the connection between medical certainties and adverse selection are still needed.

## Conclusion

The central question of our paper was whether it was possible to counteract adverse selection in the healthcare system—exacerbated by new, AI-generated medical certainties—by limiting or banning AI or its medical use, or by limiting access to medical certainties. After laying out how AI reduces existing medical uncertainties and generates new medical certainties by providing highly precise predictions of future medical conditions of individuals, we show how people can use this information to weigh the cost and benefits of switching from statutory to private health insurance and how this might lead to adverse selection and threaten public healthcare systems.

Finding existing regulatory instruments insufficient to mitigate these threats of adverse selection, we turned to the idea of banning or limiting AI and its medical use as well as limiting access to medical certainties as to counteract adverse selection. However, neither of the presented options provide adequate solutions, instead turned out to be either illiberal, paradox, impossible or unsustainable. We conclude that reinforced adverse selection as a result of AI-produced new medical certainties cannot be tackled on a symptomatic level as it is imminent in a dual healthcare system. Rather, the challenge calls for a more fundamental solution addressing the very structure of a healthcare system.

Our interdisciplinary study allows us to contribute to two current public debates. For one, we advance the discussion regarding regulatory guidance to balance promises and perils of AI application in medicine. At the same time, we provide a novel perspective on the debate regarding structural reforms of the German and comparable healthcare systems in response to the challenges it faces. In fact, our arguments posit that—given the technological revolution AI heralds—both debates must be considered simultaneously and in relation to each other in order to attain a comprehensive policy solution in either domain.

## Author contributions

UvU, MT, DE, and CLvP contributed to the final paper through conceptualizing, writing, and editing. All authors contributed to the article and approved the submitted version.

## Funding

This work has been funded by grants from the Federal Ministry of Research and Education (Grant Numbers: 01GP1905A, 01GP1905B, and 01GP1905C).

## Conflict of interest

The authors declare that the research was conducted in the absence of any commercial or financial relationships that could be construed as a potential conflict of interest.

## Publisher's note

All claims expressed in this article are solely those of the authors and do not necessarily represent those of their affiliated organizations, or those of the publisher, the editors and the reviewers. Any product that may be evaluated in this article, or claim that may be made by its manufacturer, is not guaranteed or endorsed by the publisher.

## Footnotes

^1^Some clarifications on how we understand certain terms related to healthcare systems: *Healthcare system*: Umbrella term for all institutions involved in financing medical treatment. Most follow a dual public-private structure; *Public healthcare system*: Part of the healthcare system to which all citizens belong compulsorily and which provides basic medical care; *Private healthcare system*: Part of the healthcare system which citizens can enter voluntarily by taking out additional insurance and which offers additional medical services; *Statutory health insurance*: German equivalent of the public healthcare system; *Private health insurance*: German equivalent of the private healthcare system with the difference that it is an alternative to statutory health insurance, not additional.

^2^In addition to digital twins for individuals, there are also twins for entire cities or societies (Deren et al., 2021). Similar to AI-technologies for public health surveillance, these digital twins are created from the real-time data that its members produce which is modeled into a dynamic representation of the city or society. This digital twin of society can be used, for example, to simulate how certain diseases might spread in a society or to predict trends in current infectious diseases (Kamel Boulos and Zhang, 2021). In the context of the COVID-19 pandemic, for instance, such digital twins were used to predict the spread of COVID-19 within societies (Wong, 2021)—and to simulate which measures would be most effective in containing its spread (Sahal et al., 2022). This is how digital twins have created medical certainties for public health.
